# “Tell Me How Much Your Friends Consume”—Personal, Behavioral, Social, and Attitudinal Factors Associated with Alcohol and Cannabis Use among European School Students

**DOI:** 10.3390/ijerph18041684

**Published:** 2021-02-10

**Authors:** Stefanie M. Helmer, Gregor Burkhart, João Matias, Christoph Buck, Feline Engling Cardoso, Julian Vicente

**Affiliations:** 1 Institute for Health and Nursing Science, Charité—Universitätsmedizin Berlin, Corporate Member of Freie Universität Berlin, Humboldt-Universität zu Berlin, and Berlin Institute of Health, 13353 Berlin, Germany; 2 European Monitoring Centre for Drugs and Drug Addiction, 1249-289 Lisbon, Portugal; gregor.burkhart@emcdda.europa.eu (G.B.); joao.matias@emcdda.europa.eu (J.M.); feline.engling@gmail.com (F.E.C.); julian.vicente@emcdda.europa.eu (J.V.); 3 Leibniz Institute for Prevention Research and Epidemiology—BIPS, 28359 Bremen, Germany; buck@leibniz-bips.de

**Keywords:** school, risk behavior, drunkenness, alcohol, cannabis use, prevention, descriptive norms

## Abstract

Background: Substance use in European adolescents remains a serious health concern. Assessing what affects adolescents’ substance use is crucial for implementing effective prevention. This study aims to examine alcohol and cannabis use-related behavioral, social, and attitudinal variables that might directly be considered to guide prevention responses for adolescents. Methods: Cross-sectional data of 78,554 15–16-year-old school students from the 2011 European School Survey Project on Alcohol and Other Drugs (ESPAD) from 26 European countries were analyzed. Self-reported drunkenness in the last 30 days and cannabis use in the last 12 months served as dependent variables. To investigate which factors are associated with risky substance use, multivariable logistic regressions were used. Results: 17.7% of respondents reported drunkenness in the last 30 days, and 14.9% used cannabis in the last 12 months. The most important predictor for risky substance use was the perception that most/all of their friends engaged in substance use behavior, followed by lack of parental support, low personal adherence to rules, and low school performance. Conclusion: Interventions addressing the perceived descriptive norms either directly or by changing environmental cues, opportunities, and regulations, as well as effective parenting and academic support may prevent and reduce risky substance use behavior among adolescents.

## 1. Introduction

Substance use in adolescents is a serious health concern not only in Europe, as it can lead to substance use disorders with the related harms to themselves and to society [1,2]. In order to design effective interventions, it is imperative to understand the risk and protective factors that influence young people’s substance use behavior, and which can be addressed by prevention interventions.

Reflecting on the period from 1999 to 2015, lifetime alcohol use has experienced a significant reduction amongst young people in Europe based on data from the European School Survey Project on Alcohol and other Drugs (ESPAD), collecting data from 15- to 16-year old school students. Male school students were reducing their alcohol use to a higher extent than female school students. This does not extend to heavy episodic drinking, often defined as five or more drinks on one occasion at least once in the last 30 days, which increased in girls in most European regions (except northern Europe) and in boys in southern Europe and in the Balkan region. Changes in last month cannabis use showed no clear pattern between 1999 and 2015 across European regions and sex, whereas a linear decreasing trend in cannabis use among young people in northern Europe and boys in western Europe and a decreasing concave trend in young people in eastern Europe were found; a linear increasing trend in southern Europe and an increasing convex trend in the Balkan region has been observed [3].

In the Health Behavior in School-Aged Children (HBSC) study, a significant decreasing trend in drunkenness among adolescents 11 to 15 years old was found in 23 out of 36 countries and regions for females as well as males [4]. However, the prevalence is still high among European school students [5]. A comparative analysis of data from 1997/1998 and 2005/2006 showed convergence in adolescent drunkenness across cultures and gender groups [6]. A decreasing trend in regard to risky alcohol use can also be seen outside Europe. The annually conducted Monitoring the Future Study from the US found a declining trend in alcohol use among eighth to 12th graders [7].

Faced with changes in substance use, and prevalences of high-risk behavior remaining high among European school students, questions must be asked about whether we know enough about the drivers that affect adolescents’ substance use behavior in order to design effective policies and interventions. As a first step, prevention strategies must take behavioral, social, and attitudinal determinants into account. Prevention science in the past few decades has identified descriptive and injunctive norms, parenting styles, school climate, and academic performance as well as personal and environmental characteristics as risk and protective factors [8,9]. As for descriptive norms in school populations in particular, Kuntsche and Jordan, based on a Swiss school students sample [10] and data of US school students reported by Mrug et al. [11], found perceived peer substance use (alcohol and cannabis) to be associated with corresponding personal substance use. Fletcher et al. [12] identified the peer group and desire for a sense of belonging as key influences. Sanders et al. [13] stressed the importance of perceived peer use and misperceptions of peer use, whilst Haase and Pratschke [14] emphasized self-esteem, parental concern, negative school experience, and self-selection into peer groups. Clark et al. [15], as well as Lac and Crano [16] also referred to peer group influences, whilst underlining the importance of parental monitoring and/or warmth as protective factors.

There is a broad agreement about the key factors involved, with peer influences being particularly strongly associated with both licit and illicit substance use. Such effects are assumed to work (i) via emulation of behavior by important others (in line with Social Influence Theory [17]), (ii) by peer selection and subsequent deviancy training [18] or norm-narrowing [19], (iii) via conformity with perceived norms [20,21], or (iv) by pursuit of others’ behavior and approval (Social Norms Theory [22]).

A key question is whether existing prevention strategies actually take into account what we know about behavioral, social, and attitudinal influences. To date, prevention has been conceived as universal, selective, or indicated interventions [23], and the term environmental prevention has been introduced, either as a forth form or an additional prevention function [24]: an extended classification overlaying the three forms of intervention with a classification of the function of interventions has been suggested by Foxcroft [25]. The functional types of intervention proposed by Foxcroft are environmental, developmental, and informational. In this regard, the cognitive processing is mostly secondary to behavior that emerges from the transaction between an individual and objects in their environment [25]. However, utilizing the classification of both the forms and functions of interventions results in a three-by-three matrix of nine types of interventions.

The aim of the present study was to investigate the associations between behavioral, social, as well as attitudinal factors on licit and illicit substance use that can be of interest for prevention strategies in relation to adolescent substance use. We aimed to develop recommendations for approaches to prevent adolescent substance use on the basis of a pan-European dataset.

## 2. Materials and Methods

### 2.1. Materials

The present analysis is based on the European School Survey Project on Alcohol and other Drugs (ESPAD). Data were collected through probabilistic, nationally representative ESPAD surveys of the school student population in 25 countries of the European Union (EU) (Belgium—Flanders, Bulgaria, Croatia, Cyprus, Czechia, Denmark, Estonia, Finland, France, Germany, Greece, Hungary, Ireland, Italy, Latvia, Lithuania, Malta, Netherlands, Poland, Portugal, Romania, Slovakia, Slovenia, Sweden, the United Kingdom) plus Norway from 2011. School surveys were designed to estimate substance use among school students in Europe in a most comparable way possible [26]. Data were collected from school students by means of a common, self-completed, anonymous questionnaire, following standardized methods. Analysis of school survey data for this study was facilitated by direct access to the centralized ESPAD school survey database, which provided a total sample of nearly 80,000 15–16-year-old school students surveyed in 2011.

The 2011 survey included questions on the school students’ personal use of licit and illicit substances as well as on specific risky substance use behavior. School students were also asked about their perceptions of their peers’ substance use behavior, parents’ attitudes towards drug use, personal adherence to rules, and academic performance. Demographic data, including participants’ year and month of birth, gender, and household situation were also collected. Some questions were not included in all national questionnaires, i.e., personal adherence to rules was only asked in eleven out of the analyzed 26 countries. Ethical approval procedures took place at national level, when necessary.

### 2.2. Measurements

In order to measure personal risky drinking behavior, school students were asked on how many occasions during the last 30 days (if any) they have been intoxicated from drinking alcoholic beverages, for example, staggered when walking, not being able to speak properly, throwing up, or not remembering what happened. Furthermore, school students were asked how often they engaged in heavy drinking (having had five or more drinks on one occasion in the last 30 days). As this question was not included in all national questionnaires, only drunkenness was chosen as a dependent variable for risky alcohol use. In our study, we also examined cannabis use during the last 12 months. For both dependent variables, response options ranged from “0 times” and “1–2 times”, to “40 or more times”. Dichotomous variables were created distinguishing between school students that showed risk behavior in the specific time period (were drunk at least once/used cannabis at least once) and school students who did not.

Perceptions of peer alcohol and cannabis use were assessed using the question “How many of your friends would you estimate (i) …get drunk, (ii) …smoke marijuana or hashish (cannabis)”. Response options were “None”, “A few”, “Some”, “Most”, and “All”. Five-point Likert scales were used to measure the extent to which subjects agree or disagree with the following statements about parental support: “I can easily get warmth and caring from my mother and/or father…” (1 = Almost always; 5 = Almost never) and personal adherence to rules: “I follow whatever rules I want to follow…” (1 = Totally agree; 5 = Totally disagree). Moreover, we collected data on academic performance using the question “How often during the last 12 months have you performed poorly at school or work?” Response options ranged from “0 times” and “1–2 times” to “40 or more” and were collapsed into three categories: “0 times”, “1–2 times”, and “3 times and more often”.

### 2.3. Statistical Analysis

Descriptive analysis was performed using tabulations of prevalence of individual risky alcohol and cannabis use behavior by sex and by country. Two logistic regression analyses were conducted to examine associations between independent behavioral, social, and attitudinal factors and both individual risky alcohol and cannabis use behavior. Sex, European region, perceived substance use of friends, parental support, personal adherence to rules, and school performance were included as categorical independent variables in each of the multivariable logistic regression models. Due to the large sample size, an alpha level of 0.01 was used to evaluate the significance for detected associations in each of the models [27].

Before entering the independent variables into the model, the multicollinearity between independent variables was assessed based on tolerance and VIF coefficients. Indices for both regression models indicated that no multicollinearity problem occurred. Finally, we added interaction terms between sex as well as European region and perceived peer use and adherence to rules. Stratified analyses by sex and European region were conducted. Data analysis was performed using SPSS for windows, version 26.0.

## 3. Results

Participants were 78,554 school students of the 2011 ESPAD study. Results indicate a prevalence of 17.7% risky alcohol use (drunk in the last 30 days) and 14.9% of risky cannabis use (use at least once in the last 12 months) among 15–16-year-old school students in 26 European countries (Table 1).

The perception of peer use of alcohol and cannabis was significantly associated with a higher likelihood for personal risky alcohol and cannabis use, respectively. School students who perceived that most or all of their peer school students got drunk were more likely to report getting drunk themselves (OR_Most_: 9.30, 99% CI 8.11–10.66, OR_All_: 18.07, 99% CI 15.38–21.23). With regard to cannabis use, perceived use of peers was significantly associated with reported use in the last year (OR_Most_: 97.30, 99% CI 84.16–112.49, OR_All_: 97.36, 99% CI 75.30–125.88) (Table 2). School students who reported not to experience parental support had a significantly higher chance to engage in risky alcohol (OR_Almost never_: 1.73, 99% CI 1.49–2.01) and/or cannabis use (OR_Almost never_: 1.74, 99% CI 1.46–2.07) behavior. Moreover, school students who stated they follow whatever rules they want to follow showed a higher odds for individual risky alcohol (OR: 2.40, 99% CI: 2.05–2.81) and/or cannabis use (OR: 1.83, 99 CI: 1.50–2.24) (Table 2 and Table 3).

In the alcohol use model, interaction terms showed that the effect on perceived peer use was modified only by sex, while personal adherence to rules was modified by sex and European region. In regard to cannabis use, interaction terms showed that the effect on perceived peer use was modified by sex as well as European region, whereas personal adherence to rules was modified by European region (Appendix A Table A1). A stratified analysis of perceived peer use by sex showed higher ORs for female school students than for males in regard to cannabis use and drunkenness. However, perceived peer use remained significantly associated in both groups. After stratification, adherence to rules was significantly associated with reported drunkenness in female school students. In male school students, the category closest to the reference category (rather disagree vs. totally disagree) was not significantly associated after stratification (Table 2 and Table 3). Stratified results for adherence to rules by European region resulted in a complete separation of data, since adherence to rules was asked only in Western Europe and therefore no region-specific estimate was able to be generated (Appendix A Table A1).

## 4. Discussion

Country-level rates of drunkenness (from 10% to 37%) and cannabis use (from 4% to 35%) varied widely across school students in Europe. Perceived use of friends (i.e., descriptive norms) was found to be the most important factor that was associated with personal risky alcohol and cannabis use in our sample. Several studies on substance use in European university or college students arrived at comparable results, showing that perceived peer substance use exerts high behavioral influence on emerging adults [28,29,30]. However, less research has been conducted on secondary school students in Europe. A study from Switzerland [10] was in line with our European results, finding that perceived drunkenness and cannabis use were associated with the corresponding personal use among eighth and ninth graders. Additionally, the authors report that a stronger relation between personal use and perceived peer use was found in classes where school students came cannabis-intoxicated to school or used cannabis in school premises than in schools without such incidences [10]. Such associations between the perceptions of peer use and personal use were also identified in European [31] and US adolescents [32]. The cannabis use of friends seemed to be more important among adolescents than the use of adults or siblings [33].

The fact that school students are vulnerable to their friends’ influences can be largely explained by their specific developmental stage. Adolescents become increasingly concerned with descriptive (everybody does it) and injunctive norms (everybody finds it okay) in their environment and find higher emotional reward in social conformity with such norms [34,35]. In this developmental stage, adolescents spend significantly more time with peers, and the peer group increases due to the frequent changing of classes and increased time spent out of classes with peers [36,37]. Interventions using techniques to train resistance skills against peer pressure in regard to risk health behavior might thus be a valid method for prevention, and resistance skills are included as component in so-called life-skills programs. However, simply the increase of and propensity to social cohesion during adolescence might explain increased substance use alone [38,39], without an explicit role of “peer pressure”. Several evidence-based prevention programs, such as the Unplugged study [40], focussing on developmental components (i.e., skills and competences) and normative beliefs are available in Europe, yet need larger implementation.

Furthermore, it might be of relevance to tackle possible misperceptions about peer substance use behavior. Our study showed that especially participants who perceived that their peers engaged in risky alcohol and cannabis use showed a higher likelihood to report drunkenness episodes or the use of cannabis on their own. Perceiving most or all of their peers to be risky alcohol or cannabis users seems to be an exaggerated perception—as already described in other studies [28,30]—as the prevalence estimates in ESPAD suggest that the majority does not engage in risky substance use behavior. The strong relationship of descriptive norms found with risky substance use in this analysis can be explained by two alternative theoretical frameworks with the respective options for prevention strategies, which are complementary to each other. One, in line with Social Norms Theory, argues that the (mis)perception of the behavior of relevant others (“peers”) influences own behavior, as explained above [22]. Prevention responses responding to this assumption are the so-called social norms interventions: they aim at correcting the frequent overestimation of peer substance use among school and university students [41].

In Denmark, the country that has been found to show the highest drunkenness rates among school students in our study, an intervention was tested focusing on drinking behavior in this specific target group. School students that received the intervention showed decreased overestimations of peer use and alcohol-related harms compared to control. Effects on the individual drinking behavior were only found in the sub-sample of pupils who stated that it would be okay if they drank more [42]. Overall, the evidence for the effectiveness of such approaches in Europe is still low [43], and more research among adolescents is needed. To our knowledge, there are no studies available focusing on effects of social norms interventions on cannabis use in European school students.

An alternative explanatory model virtually inverts the assumed chain of events: when school students fill in the ESPAD questionnaire, they report on their own use during the last year/last month, but also on the perceived use of their peers in the current moment. Therefore, the very procedure of data collection reflects that consumption behavior precedes the descriptive norm. The reasoning is in line with the postulation that “attitude follows behavior” [44], suggesting, in this case, that substance-using school students select their peers according to their own behavior and therefore perceive most of their peers as having risky alcohol or cannabis use (norm-narrowing). Under this scenario—where substance-users have been chosen to be peers—there is no normative fallacy to be corrected by social norms interventions. This perspective is underpinned by a recent study, where 39 participants with a cannabis disorder had to nominate close peers and estimate the level of substance use of these. The estimates were accurate, which the authors interpret as challenging the “false consensus theory”. The findings, however, rather support the hypothesis that people quite accurately assess the level of use of close peers whom they have selected themselves [45].

The adequate response principle under this hypothesis appears to be environmental prevention, which aims at reducing the opportunities for undesirable behavior (here: risky alcohol and cannabis use) to occur. This, in turn, reduces the acceptability, normality, and visibility of these behaviors and, consequently, norms, beliefs, attitudes, and values surrounding the undesired behavior also change. Complementary to skills training and the correction of cognitive fallacies, the modus operandi here is to change and sustain modified behavior—often inconspicuously—by altering the physical, economic, and regulatory aspects of the environment in which a certain behavior occurs [24]. Physical (e.g., outlet distance to schools), economic (e.g., lower prices for non-alcoholic drinks), and regulatory (e.g., substance use policies in schools and age checks in outlets) environmental prevention interventions at local level should consistently be improved.

Next to perceived substance use of peers, poor parental support was also found to be a significant risk factor for alcohol and cannabis use among school goers. This is in line with research findings suggesting that parental support belongs together with parental monitoring, parent–child relationship quality, parental support, and parental involvement to the longitudinal protective factors for both alcohol initiation and levels of later alcohol use/misuse that are modifiable, alongside with parental provision of alcohol, favorable parental attitudes towards alcohol, and parental drinking as risk factors [46]. The Monitoring the Future study conducted in the US showed that parental monitoring was one of the strongest predictors of substance use among school students, and it was found especially protective against substance use for high-risk-taking adolescents [47]. Consequently, selective preventive efforts are required. Several evidence-based interventions are available that address these factors [48,49].

Poor academic performance was found to be associated with risky alcohol as well as with cannabis use behavior in this analysis. There is additional evidence for a reciprocal association between academic performance and parental support [50], also across cultures [51]. Both factors that were examined in our study therefore suggest further implementing interventions to improve parental skills as an additional preventive strategy.

Due to the cross-sectional design, we cannot determine causal inferences between academic performance and substance use. Other studies in this field show a clear negative relation between school performance and substance use, but assume a reverse association [52,53]. A study measuring trajectories of academic self-esteem in a younger target group (13 to 15 years), however, showed that substance use is associated with persisting or increasing perceptions of academic failure. School students who perceived that their academic performance increased during the survey period or remained at a good level showed a smaller increase in substance use behavior between the survey time points [54]. In regard to cannabis, a European prospective cohort study suggested that adolescent cannabis use is not associated with educational performance, after adjustment for potential confounders such as smoking [55]. A longitudinal French study [56] showed that over three birth cohorts that less educated cannabis use initiators more often shifted to daily use than the most educated, thus giving support to academic underachievement predicting more cannabis use. Interpreting our data in the context of these additional studies warrants the need for education policies that are committed to providing particular support to school students with less academic success and hence less engagement with school. It seems important to also consider such policies as prevention interventions.

In general, it is common for schools in Europe to employ alcohol and drug policies to regulate access to substances on school grounds or events and set standards for the acceptability of alcohol and drugs in school. However, a study from the US and Australia highlighted that the means of their implementation is crucial for influencing substance use. Evans-Whipp et al. [57] found that lax policy enforcement was associated with an increased likelihood of drinking. Counselling for alcohol policy violators reduced the likelihood of risky alcohol use. However, perceptions of harsh penalties were unrelated to alcohol use.

Our study demonstrates that ESPAD provides a valuable data source to identify factors that are found to be associated with alcohol and cannabis use among adolescents and which can be targeted by prevention strategies. The multi-national perspective offers the opportunity to tailor prevention strategies to the needs of specific European regions. Morgan et al. [58] already addressed this issue when analyzing 1995 data from ESPAD across the then 26 participating countries. Morgan et al. stated that most of the research about risk factors for substance use employed individualistic research designs, only differentiating between adolescents who use various substances and those who do not. Their study results indicated that some results from the individualistic tradition are confirmed in cross-cultural comparison, while other results are less so [58].

The focus should arguably now turn to the question of how our findings might be used as a basis to improve preventive interventions in the future.

The strengths of this study are the sample size and the multinational standardized data collection. As ESPAD data are collected on a regular basis, they can give an insight in changes and trends over time. However, the findings of our study should be interpreted with several caveats. It should be noted that the German ESPAD survey covered only five of the 16 federal states, and the UK survey had low participation of invited schools in 2011. In general, results might not be fully generalizable to all school students of the participating countries. Furthermore, the same questions were not integrated into the respective questionnaires in all countries. Even though most of the questions were asked in all countries, heavy drinking and personal adherence to rules were not. We have therefore decided not to select binge drinking as a proxy for risky alcohol use. Furthermore, as the personal adherence to rules was not addressed in questionnaires in Western Europe, no European area-specific estimate for Western Europe could be generated in stratified analyses. Moreover, ESPAD is based upon self-reported data, and an under- or overestimation of risky substance use behavior due to social expectation bias cannot be ruled out. Furthermore, the ESPAD survey does not obtain information on when exactly, on what specific occasions, and which kind of alcoholic beverages were used. Thus, we cannot draw any conclusions as to whether substances were used in a nightlife context or in everyday life. This information should be included in future studies, since other studies from the US showed that assessing this kind of information (i.e., the frequency of evenings out with friends (unsupervised by adults)) regarding alcohol and substance use is important [59]. Additionally, due to the research design and the asked questions in ESPAD, we cannot say whether the perception of peer use follows personal behavior (norm-narrowing), or it is an overestimation of peer use, or both. Since the perception is the most important associated factor, it should it should be investigated in more depth in future studies.

## 5. Conclusions

This study provides intriguing results supporting the importance of perceived social norms for adolescent risky substance use and provides potential new directions for prevention strategies that combine environmental with skills-based (“developmental”) elements, with a particular need for parental interventions.

Further longitudinal studies might elucidate the still open question whether descriptive norms are a consequence of peer selection or a primary influence of behavior.

## Figures and Tables

**Table 1:** Sample characteristics and high risk alcohol and cannabis use behavior (%) in 15–16-year old school students by country.

Country	N (n = 78,554)	% Females	% Drunk in the Last 30 Days (n = 77,432)	% Cannabis Use in the Last 12 Months (n = 77,855)
Belgium	1798	45.8	11.9	20.0
Bulgaria	2217	48.9	20.4	18.5
Croatia	3002	50.7	20.8	12.5
Cyprus	4243	51.8	14.2	6.8
Czechia	3913	51.3	21.3	29.7
Denmark	2181	55.1	36.6	15.2
Estonia	2460	50.9	12.4	16.8
Finland	3744	51.5	21.2	8.5
France	2572	53.6	19.4	34.9
Germany	2796	54.0	18.3	13.9
Greece	5919	50.4	13.1	5.4
Hungary	3063	47.5	22.5	14.1
Ireland	2207	49.7	23.4	14.1
Italy	4837	49.1	12.8	18.4
Latvia	2622	49.1	17.9	16.9
Lithuania	2476	50.0	19.8	12.5
Malta	3377	50.0	19.6	7.8
Netherlands	2044	49.3	15.1	22.3
Norway	2938	49.0	14.3	4.3
Poland	5934	52.2	14.2	21.1
Portugal	1965	58.0	14.1	15.5
Romania	2770	53.8	10.4	5.6
Slovakia	2009	50.0	23.6	19.1
Slovenia	3186	51.0	20.7	19.3
Sweden	2569	49.0	13.9	6.4
United Kingdom	1712	49.5	26.5	20.6
Total	78,554	50.8	17.7	14.9

**Table 2:** Results of multivariable logistic regressions in terms of odds ratios (ORs) and 99% confidence limits (CI) to investigate the associations between individual risky alcohol use and corresponding behavioral, social, and attitudinal factors based on full sample and stratified by sex.

Variables		Drunk in the Last 30 Days (Ref.: No)											
		Model 1 (Full Sample)				Model 1.1 (Male)				Model 1.2 (Female)			
		N	%	OR	99% CI	N	%	OR	99% CI	N	%	OR	99% CI
Friends get drunk/Friends use cannabis	None	9950	13.8	1.00		4995	14.3	1.00		4955	13.4	1.00	
	A few	19,862	27.6	1.94	1.68–2.24	9783	28.0	1.75	1.45–2.10	10,079	27.2	2.28	1.80–2.89
	Some	21,966	30.5	4.04	3.52–4.63	10,616	30.4	3.60	3.03–4.29	11,350	30.6	4.86	3.88–6.09
	Most	17,303	24.0	9.30	8.11–10.66	7993	22.9	8.14	6.84–9.69	9310	25.1	11.34	9.08–14.17
	All	2978	4.1	18.07	15.38–21.23	1567	4.5	15.99	12.97–19.71	1411	3.8	21.84	16.90–28.22
Parental support	Almost always	39,750	55.2	1.00		17,649	50.5	1.00		22,101	59.6	1.00	
	Often	18,302	25.4	1.18	1.11–1.26	9955	28.5	1.14	1.05–1.25	8347	22.5	1.23	1.12–1.36
	Sometimes	8893	12.3	1.36	1.25–1.47	4727	13.5	1.35	1.21–1.51	4166	11.2	1.35	1.20–1.52
	Seldom	3247	4.5	1.67	1.49–1.88	1582	4.5	1.74	1.48–2.05	1665	4.5	1.58	1.34–1.87
	Almost never	1867	2.6	1.73	1.49–2.01	1041	3.0	1.81	1.48–2.21	826	2.2	1.60	1.27–2.01
Adherence to rules	Totally agree	6368	8.8	2.40	2.05–2.81	3790	10.8	2.05	1.67–2.53	2578	6.9	2.78	2.17–3.57
	Rather agree	7266	10.1	1.72	1.47–2.02	3605	10.3	1.40	1.13–1.74	3661	9.9	2.19	1.72–2.78
	Don’t know	10,258	14.2	1.51	1.30–1.76	4966	14.2	1.29	1.04–1.58	5292	14.3	1.83	1.45–2.32
	Rather disagree	6396	8.9	1.06	0.90–1.26	2651	7.6	0.90	0.71–1.14	3745	10.1	1.31	1.02–1.69
	Totally disagree	4799	6.7	1.00		2141	6.1	1.00		2658	7.2	1.00	
Poor school performance	Never	23,555	32.7	1.00		11,726	33.5	1.00		11,829	31.9	1.00	
	1–2 times	25,391	35.2	1.21	1.13–1.30	11,694	33.5	1.17	1.06–1.29	13,697	36.9	1.26	1.14–1.40
	3 times or more	23,113	32.1	1.70	1.58–1.82	11,534	33.0	1.58	1.44–1.73	11,579	31.2	1.84	1.67–2.04
European region	Central/Eastern Europe	24,457	33.9	1.00		11,852	33.9	1.00		12,605	34.0	1.00	
	Northern Europe	18,098	25.1	0.90	0.83–0.98	8859	25.3	0.78	0.69–0.88	9239	24.9	1.04	0.92–1.17
	Western Europe	10,528	14.6	0.87	0.79–0.96	5115	14.6	0.80	0.70–0.93	5413	14.6	0.96	0.83–1.10
	Southern Europe	18,976	26.3	0.91	0.84–0.98	9128	26.1	0.90	0.81–0.99	9848	26.5	0.93	0.83–1.14
Sex	Female	37,105	51.5	1.00		-				37,105	100.0		
	Male	34,954	48.5	1.20	1.14–1.27	34,954	100.0			-			

**Table 3:** Results of multivariable logistic regressions in terms of odds ratios (ORs) and 99% confidence limits (CI) to investigate the associations between individual risky cannabis use and corresponding behavioral, social, and attitudinal factors based on full sample and stratified by sex.

Variables		Cannabis Use in the Last 12 Months (Ref.: No)											
		Model 2 (Full Sample)				Model 2.1 (Male)				Model 2.2 (Female)			
		N	%	OR	99% CI	N	%	OR	99% CI	N	%	OR	99% CI
Friends get drunk/Friends use cannabis	None	38,585	51.8	1.00		17,938	49.6	1.00		20,647	53.9	1.00	
	A few	22,559	30.3	9.32	8.34–10.42	11,386	31.5	8.72	7.57–10.04	11,173	29.2	10.39	8.68–12.44
	Some	9779	13.1	35.63	31.79–39.93	4892	13.5	31.78	27.40–36.81	4887	12.8	42.15	35.15–50.54
	Most	2940	3.9	97.330	84.2–112.5	1525	4.2	85.38	70.25–103.75	1415	3.7	115.69	92.73–144.33
	All	591	0.8	97.36	75.30–125.88	404	1.1	73.27	53.86–99.67	187	0.5	170.16	107.04–270.50
Parental support	Almost always	41,033	55.1	1.00		18,277	50.6	1.00		22,756	59.4	1.00	
	Often	18,977	25.5	1.19	1.10–1.28	10,309	28.5	1.15	1.04–1.27	8668	22.6	1.23	1.10–1.39
	Sometimes	9213	12.4	1.42	1.29–1.56	4889	13.5	1.32	1.17–1.49	4324	11.3	1.58	1.37–1.81
	Seldom	3337	4.5	1.88	1.65–2.15	1623	4.5	1.68	1.40–2.03	1714	4.5	2.12	1.76–2.56
	Almost never	1894	2.5	1.74	1.46–2.07	1047	2.9	1.53	1.21–1.93	847	2.2	2.00	1.55–2.59
Adherence to rules	Totally agree	6386	8.6	1.84	1.50–2.24	3797	10.5	1.76	1.36–2.28	2589	6.8	1.92	1.40–2.63
	Rather agree	7340	9.9	1.75	1.43–2.12	3640	10.1	1.68	1.30–2.17	3700	9.7	1.81	1.35–2.44
	Don’t know	10,302	13.8	1.44	1.19–1.74	4973	13.8	1.34	1.04–1.74	5329	13.9	1.55	1.15–2.07
	Rather disagree	6413	8.6	1.22	0.99–1.50	2657	7.4	1.04	0.78–1.38	3756	9.8	1.44	1.06–1.95
	Totally disagree	4817	6.5	1.00		2156	6.0	1.00		2661	6.9	1.00	
Poor school performance	Never	24,377	32.7	1.00		12,135	33.6	1.00		12,242	32.0	1.00	
	1–2 times	26,217	35.2	1.27	1.17–1.39	12,094	33.5	1.23	1.10–1.38	14,123	36.9	1.33	1.16–1.51
	3 times or more	23,860	32.0	1.87	1.73–2.03	11,916	33.0	1.84	1.65–2.04	11,944	31.2	1.92	1.69–2.18
European region	Central/Eastern Europe	24,597	33.0	1.00		11,903	32.9	1.00		12,694	33.1	1.00	
	Northern Europe	18,223	24.5	0.78	0.70–0.86	8916	24.7	0.81	0.71–0.93	9307	24.3	0.73	0.63–0.85
	Western Europe	12,591	16.9	0.99	0.89–1.10	6154	17.0	0.91	0.79–1.05	6437	16.8	1.09	0.93–1.27
	Southern Europe	19,043	25.6	0.66	0.60–0.72	9172	25.4	0.70	0.62–0.79	9871	25.8	0.60	0.53–0.69
Sex	Female	38,309	51.5	1.00		36,145	100.0			-			
	Male	36,145	48.5	1.50	1.40–1.59	-	-			38,309	100.0		

## Data Availability

3rd party data: Restrictions apply to the availability of these data. Data was obtained from the ESPAD group, following an approval of a formal application and are available (http://espad.org/databases (accessed on 29 December 2020)) with the permission of the ESPAD group.

## Appendix A

**Table A1:** Main and interaction effects for drunk in the last 30 days and cannabis use in the last 12 months.

Variables			Drunk in the Last 30 Days (Ref.: No)				Cannabis Use in the Last 12 Months (Ref.: No)			
			N	%	OR	99% CI	N	%	OR	99% CI
Friends get drunk/Friends use cannabis	None		9950	13.8	1.0		38,585	51.8	1.00	
	A few		19,862	27.6	2.22	1.65–3.00	22,559	30.3	8.56	6.83–10.72
	Some		21,966	30.5	4.29	3.22–5.72	9779	13.1	33.88	26.93–42.63
	Most		17,303	24.0	7.98	6.00–10.6	2940	3.9	81.24	61.34–107.58
	All		2978	4.1	13.3	9.49–18.5	591	0.8	107.88	59.31–196.21
Parental support	Almost always		39,750	55.2	1.0		41,033	55.1	1.00	
	Often		18,302	25.4	1.19	1.11–1.26	18,977	25.5	1.20	1.111–29
	Sometimes		8893	12.3	1.35	1.25–1.47	9213	12.4	1.44	1.311.58
	Seldom		3247	4.5	1.66	1.48–1.87	3337	4.5	1.87	1.64–2.13
	Almost never		1867	2.6	1.73	1.49–2.01	1894	2.5	1.73	1.46–2.06
Adherence to rules	Totally agree		6368	8.8	3.10	2.30–4.18	6386	8.6	2.02	1.41–2.88
	Rather agree		7266	10.1	2.54	1.91–3.38	7340	9.9	1.97	1.40–2.75
	Don‘t know		10,258	14.2	2.04	1.54–2.70	10,302	13.8	1.63	1.17–2.28
	Rather disagree		6396	8.9	1.47	1.09–2.00	6413	8.6	1.48	1.04–2.10
	Totally disagree		4799	6.7	1.00		4817	6.5	1.00	
Poor school performance	Never		23,555	32.7	1.00		24,377	32.7	1.00	
	1–2 times		25,391	35.2	1.21	1.13–1.30	26,217	35.2	1.26	1.15–1.37
	3 times or more		23,113	32.1	1.72	1.60–1.84	23,860	32.0	1.85	1.70–2.01
European region	Central/Eastern Europe		24,457	33.9	1.00		24,597	33.0	1.00	
	Northern Europe		18,098	25.1	0.41	0.22–0.77	18,223	24.5	0.99	0.55–1.77
	Western Europe		10,528	14.6	0.55	0.33–0.90	12,591	16.9	0.53	0.38–0.73
	Southern Europe		18,976	26.3	0.99	0.67–1.46	19,043	25.6	0.57	0.37–0.89
Sex	Female		37,105	51.5	1.00		38,309	51.5	1.00		
	Male		34,954	48.5	2.63	1.82–3.82	36,145	48.5	2.19	1.48–3.24	
Sex * Friends get drunk/Friends use cannabis	Ref.: Female	Ref.: None			1.00				1.00		
	Male	A few			0.76	0.56–1.02			0.82	0.65–1.02
		Some			0.72	0.54–0.96			0.73	0.58–0.92
		Most			0.69	0.52–0.91			0.71	0.53–0.95
		All			0.73	0.52–1.01			0.44	0.25–0.78
European region * Friends get drunk/Friends use cannabis	Ref. Central/Eastern Europe	Ref. None			1.00				1.00	
	Northern Europe	A few			1.26	0.72–2.11			1.44	1.08–1.93
		Some			1.64	1.07–2.50			1.56	1.15–2.11
		Most			2.58	1.69–3.93			1.86	1.21–2.85
		All			3.45	2.12–5.60			0.92	0.39–2.19
	Western Europe	A few			1.24	0.79–1.99			1.59	1.14–2.22
		Some			1.68	1.01–2.81			1.59	1.13–2.24
		Most			2.43	1.46–4.04			2.07	1.37–3.12
		All			3.36	1.92–5.90			3.40	1.49–7.77
	Southern Europe	A few			0.91	0.65–1.26			1.12	0.83–1.51
		Some			1.00	0.73–1.38			1.26	0.94–1.70
		Most			1.16	0.84–1.61			1.63	1.12–2.38
		All			1.30	0.88–1.92			1.64	0.89–3.03
Sex * Personal adherence to rules	Ref.: Female	Ref.: Totally disagree			1.00						
		Totally agree			0.74	0.54–1.02			0.88	0.58–1.31
		Rather agree			0.63	0.46–0.87			0.89	0.60–1.31
		Don’t know			0.69	0.51–0.95			0.83	0.57–1.22
		Rather disagree			0.67	0.47–0.95			0.72	0.47–1.09
European region * Personal adherence to rules	Ref. Central/Eastern Europe	Ref.: Totally disagree			1.00						
	Northern Europe	Totally agree			0.88	0.47–1.64			0.93	0.47–1.85
		Rather agree			1.22	0.67–2.23			0.94	0.49–1.81
		Don’t know			1.23	0.69–2.18			0.93	0.50–1.74
		Rather disagree			1.20	0.65–2.21			1.04	0.54–1.98
	Western Europe	Totally agree			NA	NA			NA	NA
		Rather agree			NA	NA			NA	NA
		Don’t know			NA	NA			NA	NA
		Rather disagree			NA	NA			NA	NA
	Southern Europe	Totally agree			0.82	0.59–1.14			1.07	0.69–1.65
		Rather agree			0.66	0.47–0.93			0.95	0.62–1.47
		Don’t know			0.71	0.49–1.04			1.01	0.66–1.57
		Rather disagree			0.71	0.49–1.04			0.93	0.59–1.50

* Interaction term between two variables.

## References

[1] Marshall E.J. (2014). Adolescent Alcohol Use: Risks and Consequences. Alcohol Alcohol..

[2] Morin J.-F.G., Afzali M.H., Bourque J., Stewart S.H., Séguin J.R., O’Leary-Barrett M., Conrod P.J. (2019). A Population-Based Analysis of the Relationship between Substance Use and Adolescent Cognitive Development. Am. J. Psychiatry.

[3] Kraus L., Seitz N.N., Piontek D., Molinaro S., Siciliano V., Guttormsson U., Arpa S., Monshouwer K., Leifman H., Vicente J. (2018). ‘Are the Times A-Changin’? Trends in adolescent substance use in Europe. Addiction.

[4] Canale N., Inchley J., Inchley J.C., Currie D.B., Vieno A., Torsheim T., Ferreira-Borges C., Weber M., Barnekow V., Breda J. (2018). Trends in drunkenness by gender and subregion. Adolescent Alcohol-Related Behaviours: Trends and Inequalities in the WHO European Region, 2002–2014.

[5] Moor I., Heilmann K., Hinrichs R., Richter M. (2019). Is alcohol out? Evidence from the Health Behaviour in School-aged Children (HBSC)-study. Public Health Forum.

[6] Kuntsche E., Kuntsche S., Knibbe R., Simons-Morton B., Farhat T., Hublet A., Bendtsen P., Godeau E., Demetrovics Z. (2011). Cultural and gender convergence in adolescent drunkenness: Evidence from 23 European and North American countries. Arch. Pediatr. Adolesc. Med..

[7] Patrick M.E., Schulenberg J.E. (2013). Prevalence and Predictors of Adolescent Alcohol Use and Binge Drinking in the United States. Alcohol Res. Curr. Rev..

[8] Baggio S., Mohler-Kuo M., Dupuis M., Henchoz Y., Studer J., N’Goran A., Gmel G. (2016). Substance use capital: Social resources enhancing youth substance use. Rev. D’épidémiol. Santé Publique.

[9] Simons-Morton B.G., Haynie D.L., Crump A.D., Eitel P., Saylor K.E. (2001). Peer and Parent Influences on Smoking and Drinking among Early Adolescents. Health Educ. Behav..

[10] Kuntsche E., Jordan M.D. (2006). Adolescent alcohol and cannabis use in relation to peer and school factors. Results of multilevel analyses. Drug Alcohol Depend..

[11] Mrug S., Gaines J., Su W., Windle M. (2010). School-Level Substance Use: Effects on Early Adolescents’ Alcohol, Tobacco, and Marijuana Use. J. Stud. Alcohol Drugs.

[12] Fletcher A., Bonell C., Sorhaindo A., Strange V. (2009). How might schools influence young people’s drug use? Development of theory from qualitative case-study research. J. Adolesc. Health Off. Publ. Soc. Adolesc. Med..

[13] Sanders A., Stogner J., Miller B.L. (2013). Perception vs. reality: An investigation of the misperceptions concerning the extent of peer novel drug use. J. Drug Educ..

[14] Haase T., Pratschke J. (2010). Risk and Protective Factors for Substance Use among Young People: A Comparative Study of Early School-Leavers and School-Attending Students.

[15] Clark H.K., Shamblen S.R., Ringwalt C.L., Hanley S. (2012). Predicting high risk adolescents’ substance use over time: The role of parental monitoring. J. Prim. Prev..

[16] Lac A., Crano W.D. (2009). Monitoring Matters: Meta-analytic review reveals the reliable linkage of parental monitoring with adolescent marijuana use. Perspect. Psychol. Sci..

[17] Bandura A. (1978). Social Learning Theory of Aggression. J. Commun..

[18] Rorie M., Gottfredson D.C., Cross A., Wilson D., Connell N.M. (2011). Structure and deviancy training in after-school programs. J. Adolesc..

[19] Killeya-Jones L.A., Costanzo P.R., Malone P., Quinlan N.P., Miller-Johnson S. (2007). Norm-narrowing and self- and other-perceived aggression in early-adolescent same-sex and mixed-sex cliques. J. Sch. Psychol..

[20] Cialdini R.B., Reno R.R., Kallgren C.A. (1990). A focus theory of normative conduct: Recycling the concept of norms to reduce littering in public places. J. Personal. Soc. Psychol..

[21] Elek E., Miller-Day M., Hecht M.L. (2006). Influences of Personal, Injunctive, and Descriptive Norms on Early Adolescent Substance use. J. Drug Issues.

[22] The Social Norms Approach: Theory, Research, and Annotated Bibliography. http://www.alanberkowitz.com/articles/social_norms.pdf.

[23] Gordon R. (1983). An operational classification of disease prevention. Public Health Rep..

[24] Oncioiu S.I., Burkhart G., Calafat A., Duch M., Perman-Howe P., Foxcroft D.R. (2018). Environmental Substance Use Prevention Interventions in Europe.

[25] Foxcroft D.R. (2014). “Form ever follows function. This is the law”. A prevention taxonomy based on a functional typology. Adicciones.

[26] Hibell B., Guttormsson U., Ahlström S., Balakireva O., Kokkevi T.B.A., Kraus L. (2012). The 2011 ESPAD Report. Substance Use among Students in 36 European Countries.

[27] Li X., Zhou Y., Stanton B. (2002). Illicit drug initiation among institutionalized drug users in China. Addiction.

[28] Dempsey R.C., McAlaney J., Helmer S.M., Pischke C.R., Akvardar Y., Bewick B.M., Fawkner H.J., Guillen-Grima F., Stock C., Vriesacker B. (2016). Normative Perceptions of Cannabis Use Among European University Students: Associations of Perceived Peer Use and Peer Attitudes With Personal Use and Attitudes. J. Stud. Alcohol Drugs.

[29] Helmer S.M., Sebena R., McAlaney J., Petkeviciene J., Salonna F., Lukács A., Mikolajczyk R.T. (2016). Perception of High Alcohol Use of Peers Is Associated With High Personal Alcohol Use in First-Year University Students in Three Central and Eastern European Countries. Subst. Use Misuse.

[30] McAlaney J., Helmer S.M., Stock C., Vriesacker B., Van Hal G., Dempsey R.C., Akvardar Y., Salonna F., Kalina O., Guillen-Grima F. (2015). Personal and Perceived Peer Use of and Attitudes Toward Alcohol Among University and College Students in Seven EU Countries: Project SNIPE. J. Stud. Alcohol Drugs.

[31] Piontek D., Kraus L., Bjarnason T., Demetrovics Z., Ramstedt M. (2013). Individual and country-level effects of cannabis-related perceptions on cannabis use. A multilevel study among adolescents in 32 European countries. J. Adolesc. Health Off. Publ. Soc. Adolesc. Med..

[32] Roditis M.L., Delucchi K., Chang A., Halpern-Felsher B. (2016). Perceptions of social norms and exposure to pro-marijuana messages are associated with adolescent marijuana use. Prev. Med..

[33] Schuler M.S., Tucker J.S., Pedersen E.R., D’Amico E.J. (2019). Relative influence of perceived peer and family substance use on adolescent alcohol, cigarette, and marijuana use across middle and high school. Addict. Behav..

[34] Blakemore S.-J., Robbins T.W. (2012). Decision-making in the adolescent brain. Nat. Neurosci..

[35] Chein J., Albert D., O’Brien L., Uckert K., Steinberg L. (2010). Peers increase adolescent risk taking by enhancing activity in the brain’s reward circuitry. Dev. Sci..

[36] Lam C.B., McHale S.M., Crouter A.C. (2014). Time with Peers from Middle Childhood to Late Adolescence: Developmental Course and Adjustment Correlates. Child Dev..

[37] Windle M., Spear L.P., Fuligni A.J., Angold A., Brown J.D., Pine D., Smith G.T., Giedd J., Dahl R.E. (2008). Transitions into Underage and Problem Drinking: Developmental Processes and Mechanisms Between 10 and 15 Years of Age. Pediatrics.

[38] MacArthur G.J., Jacob N., Pound P., Hickman M., Campbell R. (2017). Among friends: A qualitative exploration of the role of peers in young people’s alcohol use using Bourdieu’s concepts of habitus, field and capital. Soc. Health Illn..

[39] Martins J.G., De Paiva H.N., Paiva P.C.P., Ferreira R.C., Pordeus I.A., Zarzar P.M., Kawachi I. (2017). New evidence about the “dark side” of social cohesion in promoting binge drinking among adolescents. PLoS ONE.

[40] Vigna-Taglianti F.D., Galanti M.R., Burkhart G., Caria M.P., Vadrucci S., Faggiano F. (2014). “Unplugged,” a European school-based program for substance use prevention among adolescents: Overview of results from the EU-Dap trial. New Dir. Youth Dev..

[41] McAlaney J., Bewick B., Hughes C. (2010). The international development of the ‘Social Norms’ approach to drug education and prevention. Drugs Educ. Prev. Policy.

[42] Vallentin-Holbech L., Rasmussen B.M., Stock C. (2018). Effects of the social norms intervention The GOOD Life on norm perceptions, binge drinking and alcohol-related harms: A cluster-randomised controlled trial. Prev. Med. Rep..

[43] Foxcroft D., Moreira M.T., Santimano N.M.L.A., A Smith L. (2015). Social norms information for alcohol misuse in university and college students. Cochrane Database Syst. Rev..

[44] Jhangiani R., Tarry H., Stangor C. (2014). Changing Attitudes by Changing Behavior. Principles of Social Psychology.

[45] Mason M.J., Brown A., Moore M. (2019). The accuracy of young adult cannabis users’ perceptions of friends’ cannabis and alcohol use. Addict. Behav..

[46] Yap M.B.H., Cheong T.W.K., Zaravinos-Tsakos F., Lubman D.I., Jorm A.F. (2017). Modifiable parenting factors associated with adolescent alcohol misuse: A systematic review and meta-analysis of longitudinal studies. Addiction.

[47] Dever B.V., Schulenberg J.E., Dworkin J.B., O’Malley P.M., Kloska D.D., Bachman J.G. (2012). Predicting Risk-Taking With and Without Substance Use: The Effects of Parental Monitoring, School Bonding, and Sports Participation. Prev. Sci..

[48] Bröning S., Kumpfer K.L., Kruse K., Sack P.-M., Schaunig-Busch I., Ruths S., Moesgen D., Pflug E., Klein M., Thomasius R. (2012). Selective prevention programs for children from substance-affected families: A comprehensive systematic review. Subst. Abus. Treat. Prev. Policy.

[49] Fulkerson J.A., Pasch K.E., Perry C.L., Komro K. (2008). Relationships Between Alcohol-related Informal Social Control, Parental Monitoring and Adolescent Problem Behaviors among Racially Diverse Urban Youth. J. Community Health.

[50] Roebroek L., Koning I.M. (2015). The Reciprocal Relation between Adolescents’ School Engagement and Alcohol Consumption, and the Role of Parental Support. Prev. Sci..

[51] Degarmo D.S., Martinez C.R. (2006). A Culturally Informed Model of Academic Well-Being for Latino Youth: The Importance of Discriminatory Experiences and Social Support. Fam. Relat..

[52] Mekonen T., Fekadu W., Mekonnen T.C., Workie S.B. (2017). Substance Use as a Strong Predictor of Poor Academic Achievement among University Students. Psychiatry J..

[53] Cox R.G., Zhang L., Johnson W.D., Bender D.R. (2007). Academic Performance and Substance Use: Findings from a State Survey of Public High School Students. J. Sch. Health.

[54] Bergen H.A., Martin G., Roeger L., Allison S. (2005). Perceived academic performance and alcohol, tobacco and marijuana use: Longitudinal relationships in young community adolescents. Addict. Behav..

[55] Mokrysz C., Landy R., Gage S.H., Munafo M.R., Roiser J.P., Curran H.V. (2016). Are IQ and educational outcomes in teenagers related to their cannabis use? A prospective cohort study. J. Psychopharmacol..

[56] Legleye S., Khlat M., Mayet A., Beck F., Falissard B., Chau N., Peretti-Watel P. (2016). From cannabis initiation to daily use: Educational inequalities in consumption behaviours over three generations in France. Addiction.

[57] Evans-Whipp T.J., Plenty S.M., Catalano R.F., Herrenkohl T.I., Toumbourou J.W. (2013). The impact of school alcohol policy on student drinking. Health Educ. Res..

[58] Morgan M., Hibell B., Andersson B., Bjarnason Þ., Kokkevi A., Narusk A., Morgan B.H.M. (1999). The ESPAD Study: Implications for prevention. Drugs Educ. Prev. Policy.

[59] Patrick M.E., Schulenberg J.E. (2010). Alcohol Use and Heavy Episodic Drinking Prevalence and Predictors Among National Samples of American Eighth-and Tenth-Grade Students. J. Stud. Alcohol Drugs.

